# Effects of standard and explicit cognitive bias modification and computer-administered cognitive-behaviour therapy on cognitive biases and social anxiety^☆^

**DOI:** 10.1016/j.jbtep.2013.12.002

**Published:** 2014-06

**Authors:** Sirous Mobini, Bundy Mackintosh, Jo Illingworth, Lina Gega, Peter Langdon, Laura Hoppitt

**Affiliations:** a School of Psychology, University of Newcastle, Callaghan, NSW 2308, Australia; b Department of Psychological Sciences, Norwich Medical School, University of East Anglia, Norwich NR4 7TJ, UK; c School of Social Work and Psychology, Norwich Medical School, University of East Anglia, Norwich NR4 7TJ, UK

**Keywords:** Cognitive bias modification, Social anxiety, Computer-administered CBT, Cognitive biases

## Abstract

**Background and objectives:**

This study examines the effects of a single session of Cognitive Bias Modification to induce positive Interpretative bias (CBM-I) using standard or explicit instructions and an analogue of computer-administered CBT (c-CBT) program on modifying cognitive biases and social anxiety.

**Methods:**

A sample of 76 volunteers with social anxiety attended a research site. At both pre- and post-test, participants completed two computer-administered tests of interpretative and attentional biases and a self-report measure of social anxiety. Participants in the training conditions completed a single session of either standard or explicit CBM-I positive training and a c-CBT program. Participants in the Control (no training) condition completed a CBM-I neutral task matched the active CBM-I intervention in format and duration but did not encourage positive disambiguation of socially ambiguous or threatening scenarios.

**Results:**

Participants in both CBM-I programs (either standard or explicit instructions) and the c-CBT condition exhibited more positive interpretations of ambiguous social scenarios at post-test and one-week follow-up as compared to the Control condition. Moreover, the results showed that CBM-I and c-CBT, to some extent, changed negative attention biases in a positive direction. Furthermore, the results showed that both CBM-I training conditions and c-CBT reduced social anxiety symptoms at one-week follow-up.

**Limitations:**

This study used a single session of CBM-I training, however multi-sessions intervention might result in more endurable positive CBM-I changes.

**Conclusions:**

A computerised single session of CBM-I and an analogue of c-CBT program reduced negative interpretative biases and social anxiety.

## Highlights

• Standard and Explicit Cognitive Bias Modification for interpretative biases (CBM-I).
• Computer-administered CBT (c-CBT) for social anxiety as an alternative intervention.
• Standard and explicit CBM-I and c-CBT increased positive interpretations.
• Explicit instructions did not enhance CBM-I effects on interpretative biases.
• CBM-I training and c-CBT reduced social anxiety at one-week follow-up.

## 1 Introduction

Cognitive-behavioural models of social anxiety (Beck, Emery, & Greenberg, 1985; Clark & Wells, 1995; Rapee & Heimberg, 1997) propose that negative self-appraisals in social situations influence the development and maintenance of social anxiety. These negative appraisals may result from elaborative processing of negative information including biases in attention, interpretation, judgement, and memory (Clark & McManus, 2002; Heinrichs & Hofmann, 2001; Hirsch & Clark, 2004; Ledley & Heimberg, 2006; Musa & Lépine, 2000). A wealth of literature suggests that socially-anxious individuals are more likely to attend to social threat information (see Bögels & Mansell, 2004; Schultz & Heimberg, 2008, for reviews). Similarly, a number of studies reported that socially-anxious individuals interpret ambiguous social information in a negative or less positive manner (Amir, Beard, & Bower, 2005; Hertel, Brozovich, Joormann, & Gotlib, 2008; Huppert, Pasupuleti, Foa, & Mathews, 2007; Stopa & Clark, 2000). Given that social anxiety is associated with negative cognitive biases, a next phase of research is to establish whether such biases are amenable to modifications or treatment (Mobini, Reynolds, & Mackintosh, 2013).

Cognitive Bias Modification for interpretative biases (CBM-I) method is a text-based computerised task aimed at systematically training individuals to interpret emotionally ambiguous information in a particular direction (Mathews & Mackintosh, 2000). Subsequently, laboratory studies have developed a number of CBM interventions to directly modify cognitive biases associated with anxiety via repeated practice on computerised cognitive tasks (see Beard, 2011). This line of research suggests that it is possible to experimentally manipulate (or ‘train’) interpretation bias in healthy volunteers (e.g., Mackintosh, Mathews, Yiend, Ridgeway, & Cook, 2006; Mathews & Mackintosh, 2000; Yiend, Mackintosh, & Mathews, 2005). So far, a few published studies have used CBM procedures to modify interpretation biases in participants who have social anxiety (e.g., Beard & Amir, 2008; Murphy, Hirsch, Mathews, Smith, & Clark, 2007; Turner, Hoppitt, Hodgekin, Mackintosh, & Fowler, 2011). The results of these studies have shown that it is possible to induce positive interpretations in socially-anxious individuals and reduce social anxiety symptoms.

Although these CBM studies have reported successful modifications of interpretative bias, the method invariably avoids providing explicit instructions about the intention of the training. Instead, repeated practice is provided in which participants are guided to take a particular interpretation and they typically are not explicitly informed of the training contingency. This is based on the assumption that the training contingency is registered standardly and exerts an incidental impact on processing selectivity (Mathews & Mackintosh, 2000). If CBM-I operates at a more habitual level, then it may be resistant to manipulation by verbal instructions. Despite anticipating a continuing role for incidental learning in many CBM variants, it may be likely that the judicious use of explicit instructions at times will enhance conscious processing of the training materials, and hence enhancing CBM efficacy. A few recent studies using different variants of CBM with explicit instructions reported beneficial effects of CBM delivery (Krebs, Hirsch, & Mathews, 2010; Schartau, Dalgleish, & Dunn, 2009; Watkins, Baeyens, & Read, 2009). Watkins et al. (2009) explicitly told participants with dysphoria that the training exercises were designed to reduce their negative thinking and thereby reduce depression. Participants in Schartau et al.'s (2009) study were required to systematically practice appraising events so as to reduce the negative affect they experienced. Similarly, in the present study participants in the explicit CBM-I condition were made aware of the intention of the CBM positive training, however, unlike two previous studies they were not asked to practice the training materials at home (Watkins et al., 2009) or transfer what they learned to day-to-day experience (Schartau et al., 2009). More recently, using an attentional variant of CBM Krebs and colleagues (Krebs et al., 2010) found that providing participants with explicit instructions about the relationship between word valence and target location in a dot-probe task resulted in more effective attention modification.

Nevertheless, it should be noted that directly instructing participants to process information in a certain way could have a paradoxical effect (Wegner & Schneider, 2003). Consistent with this view, it is suggested that explicit instructions to selectively avoid specific categories of information may sometimes be unhelpful in CBM approaches (MacLeod, Martinez, & Williams, 2009). However, the validity of this assumption has not yet been tested adequately by a direct comparison between standard and explicit instructions in CBM-I training. It is not yet clear whether the provision of explicit instructions about the purpose of the training in the CBM-I paradigm developed by Mathews and Mackintosh (2000) would enhance or compromise the training effects on interpretative bias and social anxiety. The first aim of this study was, therefore, to assess the effects of standard vs. explicit instructions methods of the CBM-I training on interpretive bias and social anxiety in a high socially-anxious anxious sample.

There is a juxtaposition between the CBM-I approach which operates through standard learning and other established therapies, such as cognitive-behaviour therapy (CBT), in which patients are made consciously aware of the thought–emotion link as a mechanism for therapeutic change and are encouraged to actively engage in modifying unhelpful thinking and misinterpretations in order to feel less anxious or depressed. Franklin and colleagues found that group CBT reduced negative interpretation bias in socially phobic individuals (Franklin, Huppert, Langner, Leiberg, & Foa, 2005). However, no study has yet compared two versions of CBM-I methods (standard vs. explicit) and CBT in modification of cognitive biases in social anxiety. For the purpose of comparing CBM-I approach with a more explicit therapeutic intervention, an analogue of computer-administered CBT (c-CBT) program was developed and used in this study. Thus, the second aim of the present study was to investigate whether CBM-I induced changes are comparable with a treatment method whereby participants were directly educated about and subsequently made fully aware of the role of negative thinking in causing and maintaining social anxiety.

A third and final aim of the study was to examine whether any positive effects caused by CBM-I training or c-CBT are specific to interpretation biases or these changes can also affect the earlier stage of information processing, i.e., attention allocation. Beck and Clark (1997) introduced a three-stage model of information processing of anxiety consisting of (a) initial registration, (b) immediate preparation, and (c) secondary elaboration. It is assumed that attentional bias operates at the early stages of information processing which are responsible for initial orienting to, and rapid detection of, threat in the environment (Beck & Clark, 1997; McNally, 1995). In contrast, interpretative bias appears to operate at the later stages of information processing, possibly ‘immediate preparation’, which are responsible for interpreting and judging about the threat in the environment. Thus, the recognition of a negative stimulus leads to the immediate preparation stage involving the activation of the primal mode. Beck and Clark (1997) suggested that automatic anxious thoughts and biased cognitive processing result from the activation of the orienting and primal threat modes at the earlier stages of information processing. One of the questions to be investigated is whether modification of one of the cognitive processes, i.e., attention or interpretation, can result in changes in the other aspect of cognitive processing. In a study, White and colleagues found that individuals trained to attend to threat were more likely than individuals in a placebo training group to interpret ambiguous information in a threat-related manner (White, Suway, Pine, Bar-Haim, & Fox, 2011). These data suggest that the preferential allocation of attention towards threat in the initial stages of information may result in a cascade of subsequent processing biases. However, it is not clear whether modification of negative interpretative biases can deactivate negative attentional deployment. Thus, bias modification procedures targeting both attention and interpretation biases would have clinical implications for the treatment of anxiety disorders (see Mobini & Grant, 2007; Mobini et al., 2013). One can assume that developing more benign and positive interpretations of ambiguous situations may modify the orienting mode towards threat and reduce negative attentional biases.

To our knowledge, no study has yet compared the effects of two methods of CBM-I (standard vs. explicit instructions) and an analogue of computer-administered CBT (c-CBT) on modifying cognitive biases in social anxiety. Taken together, using experimental methodology, the present study aims to investigate three interesting research questions: 1) whether providing explicit instructions in CBM-I training can enhance its effects on interpretative biases over the standard CBM-I training, 2) whether the standard or explicit CBM-I positive training is more (or less) effective, if any, than the computer-administered CBT (c-CBT) program in reducing cognitive biases and social anxiety, and 3) finally whether any changes in interpretative bias after the treatment conditions can lead to changes in attentional biases. In general, it was hypothesised that both standard and explicit CBM-I training programs would reduce a negative interpretation bias and social anxiety in socially-anxious participants and that providing explicit instructions about the purpose of training would enhance the positive effects of CBM-I training. Also, we hypothesised that similar to CBM-I conditions, the c-CBT program would reduce a negative interpretation bias and social anxiety. Finally, it was hypothesised that positive changes in interpretative biases would decrease attentional bias towards negative stimuli.

## 2 Material and methods

### 2.1 Participants

Seventy-six (*N* = 76) socially-anxious volunteers aged between 18 and 60 who were recruited from the student and staff populations at the University of East Anglia (UEA, United Kingdom) took part in this study (see Table 1 for demographic information).

### 2.2 Design

The design of the study was a mixed experimental design with type of treatment (Standard CBM-I, Explicit CBM-I, c-CBT, and Control no training CBM-I) a between-subjects factor and measures of attentional and interpretative biases and social anxiety scores in three time intervals (pre-test, post-test and one-week follow-up) as within-subject factors. Participants were allocated into the training and control conditions through the computer-generated randomisation procedure.

### 2.3 Social anxiety assessment

*Fear of Negative Evaluation Scale* (FNE, Watson & Friend, 1969). The FNE comprises 30 true–false items that refer to expectation and distress related to negative evaluation from others in social situations. It demonstrates good internal consistency (*α* = 0.88 − 0.94) and test–retest reliability over a 1-week period (*r* = 0.94) as well as good discriminative validity for social anxiety (Oei, Kenna, & Evans, 1991). Participants who scored 17 or higher on the FNE were invited to take part in the study. A cut-off score of 17 on the FNE has been shown to be associated with high social anxiety (Stopa & Clark, 2001).

### 2.4 Interpretative bias assessment

The interpretation bias was assessed individually using a text-based encoding task in which participants were presented with a number of ambiguous social scenarios and their interpretation of these passages was assessed. This task has been widely used in a number of studies investigating interpretative biases in anxiety (e.g., Hertel et al., 2008; Mackintosh et al., 2006; Mathews & Mackintosh, 2000). A recent study demonstrated that the interpretative bias task is capable of differentiating between high and low levels of neuroticism (Salemink & van den Hout, 2010).

Participants were presented with 15 ambiguous social scenarios and instructed to imagine themselves in the situation while reading each description as if they were actually there. Each scenario ended ambiguously to allow participants to apply their own spontaneous interpretation to the meaning of the passage. After participants read all 15 scenarios, they were presented with identifying title of each ambiguous scenario along with four interpretations in an individually randomised order representing four different interpretations of each previously presented ambiguous scenario, one at a time. Two of these four sentences were target sentences matches in meaning the positive and the negative potential interpretation of the preceding ambiguous scenario. The remaining two were foils, which did not correspond to either possible interpretation of this preceding ambiguity but were positively and negatively valenced. Foils were included to assess any wider priming effects of training indicating a potential response bias for endorsing any information of a certain emotional valence. Participants were asked to rate each sentence according to how closely it corresponded in meaning to what was described in the preceding scenario. They made this rating using a 4-point Likert scale ranging from 1 (*very different in meaning*) to 4 (*very similar in meaning*). Three different versions of interpretation bias measure were used over 3 phases, pre and post-tests and follow-up, in a counterbalanced order.

### 2.5 Attentional bias task

The dot probe task was used to measure attentional bias. This task is based on the idea that subjects tend to be faster to respond to a probe stimulus (e.g., a small dot) that is presented in an attended, rather than unattended, region of a visual display (MacLeod, Mathews, & Tata, 1986). For the purpose of this study, a total of 108 word pairs, with members matched in terms of letter length and frequency of usage, were selected from a larger initial pool which included word pairs taken from similar studies (e.g., Asmundson & Stein, 1994; Pishyar et al., 2004). Each set of the dot probe task consisted of 36 word pairs. For each set a threat word was paired with its corresponding neutral word to make 12 negative-neutral pairs (e.g., boring vs. billet) and 12 physical sensation-neutral word pairs (e.g., vomiting vs. modified). Similarly, each positive word was paired with its corresponding neutral word to make 12 positive-neutral word pairs (e.g., hopeful vs. harvest).

Each trial commenced with the 500-ms central presentation of the fixation cue “+++.” Immediately following termination of this display, the target and neutral members of a word pair were presented simultaneously for 500 ms, one just above and one just below the fixation position. The word pairs appeared in the middle of the computer screen with a 3 cm vertical distance between the two words and the instructed viewing distance of 60 cm. The target word appeared with equal probability in the upper screen location or lower screen location. Upon the termination of this word pair display, one of two probes, a small symbol pointing either to the left (>) or to the right (<), immediately appeared in the location previously occupied by a randomly determined character in either word. These signs remained on the screen until the participant pressed the key. In the 8 practice trials presented at the start of the task, participants received feedback (Correct or Incorrect) for their responses. Probe identity was determined randomly on each trial. Participants were required to discriminate probe identity as quickly as possible, by pressing the “z” key if it points to the left and the “m” key if it points to the right. Participants were instructed to use their right hand to press “m” and left hand to press “z” keys on a keyboard. The probe stimulus remained on the screen until the programme detected this response, after which the screen was cleared, and the next trial commenced 500 ms later.

### 2.6 Cognitive bias modification to induce positive interpretation (CBM-I)

CBM-I method was a text-based computerised task in which participants were trained during a number of trials to consistently resolve ambiguous social situations in favour of either positive or neutral outcomes via completion of word stems (Mathews & Mackintosh, 2000; Murphy et al., 2007). The CBM-I positive training group had 100 passages (ambiguous social scenarios related to social interactions and social performance) that were four lines in length. Participants were instructed to imagine themselves in the situation while reading each passage as if they were actually there. This is in line with Holmes and colleagues findings that mental imagery is more effective than verbal training in inducing positive mood (Holmes, Mathews, Dalgleish, & Mackintosh, 2006). The passages were designed to stay emotionally ambiguous until the last word (presented as a fragment, e.g., *fri--d-y*), that would always disambiguate the passage in a positive way (*friendly*).

In contrast to the standard CBM-I condition in which participants were given the usual minimal instructions, in explicit CBM-I participants were explicitly instructed that they would see emotionally ambiguous scenarios, and to do well on the task they should try and resolve the scenarios in a positive way. While the general instructions were identical in both CBM-I programs, the following instruction was provided for the participants in the explicit CBM-I group: “*In the main task, each situation will begin emotionally ambiguously but turn out well in the end (like the final practice item). If you bear in mind that all the situations end positively this will help you with the task.*”

### 2.7 Control no training condition

In this control (no training) condition, the passages were similar to those used in the CBM-I training condition, with the critical exception that these passages did not communicate ambiguous scenarios, amenable to positive or negative interpretation, but unambiguous scenarios that are all emotionally neutral in tone.

### 2.8 Computer-administered CBT for social anxiety (c-CBT)

The c-CBT program used in this study was developed based on cognitive models of social anxiety (Beck et al., 1985; Clark & Wells, 1995; Rapee & Heimberg, 1997). The self-help materials were adapted from self-help CBT-based guidebooks for social anxiety (Antony & Swinson, 2008; Butler, 2008) and modified for use via computer in a very condensed way. This program was revised based on the feedback from a pilot study with a small number of volunteers prior to using in the main study. The final c-CBT program was presented as 55 PowerPoint slides on the PC monitor in text format with relevant visual prompts on each slide to encourage self-reflection. The c-CBT program was an alternative intervention to compare the CBM-I effects with an analogue of computerised CBT.

The c-CBT for social anxiety was comprised of 3 main parts including 1) psychoeducation about CBT and social anxiety, 2) socialising to CBT model of social anxiety and the role of anxiety-provoking thoughts, assumptions and core beliefs in causing and maintaining social anxiety, and 3) overcoming social anxiety using behavioural and cognitive strategies. Throughout the program participants were encouraged to reflect on their cognitions and identify their negative automatic thoughts in social situations using an imagery method. They were taught about the role of cognitive distortions in avoidance behaviour and how their fear of social situations can be maintained. The maintenance cycle was discussed along with some behavioural and cognitive strategies to break this cycle. Each part ended with a quiz consisting of seven relevant questions for participants to answer. The aim of these quizzes was to encourage participants to concentrate on the training materials and consolidate their learning; in answering these quizzes they were told that this is not a test of their learning. The duration of completion of this program was approximately 45–50 min.

## 3 Results

The participants' demographic information for each group is depicted in Table 1. The four groups did not differ from each other in terms of age, *F*(3,73) = .29, *p* = .66 and as shown in the Table 1 gender and ethnicity of the participants were evenly distributed across four study groups.

### 3.1 Interpretation bias

Participants' recognition ratings of disambiguated versions of the final sentences of the test scenarios were the main measure of interest to show the persistence of any training effects. The mean ratings for each participant were calculated across the four different sentence type: negative target, positive target, negative foil, and positive foil. Three bias scores for each participant (Pre-test, Post-test, one-week follow-up) were calculated by subtracting the mean recognition rating for the negative targets from the mean recognition ratings for the positive targets. This gave each participant three bias scores that could range from −3 to +3, with a negative score indicating a negative bias and a positive score representing a positive bias. Similar bias scores were calculated by subtracting the mean recognition rating for the negative foils from the mean recognition ratings for the positive foils.

Table 2 summarises the bias scores for target and foil sentences (positive and negative) for each group at pre-test, post-test and one-week follow-up. It was hypothesised that socially-anxious individuals in the CBM-I and c-CBT conditions would demonstrate an increase in positive interpretations (and reduction in negative interpretations) of ambiguous scenarios as compared to the control condition. To test this hypothesis, the mean values of bias scores were entered into an omnibus three-way mixed ANOVA with Group (standard CBM-I, explicit CBM, c-CBT, Control) as a between-subjects factor and Time (pre-test, post-test, and follow-up), Sentence Type (targets vs. foils), as within-subjects factors. The results indicated a significant three-way interaction effect, Time × Sentence Type × Group, *F*(6,144) = 2.17, *p* < .05. To follow up this interaction and investigate group differences, the bias change scores were subjected to one-way analysis of variance (ANOVA). The results showed significant group differences for target sentences from pre-test to post-test, *F*(3,73) = 2.87, *p* < .05, and from pre-test to one-week follow-up *F*(3,73) = 3.10, *p* < .05. The change scores were then subjected to post-hoc comparisons using the Fisher's Least Significant Difference (LSD). The results revealed significant group differences on targets from pre-test to post-test between Standard CBM-I and Control, *t*(37) = 2.20, *p* < .05, *d* = 1.12 , Explicit CBM-I and Control *t*(37) = 2.62, *p* < .01, *d* = 1.88, and c-CBT and Control, *t*(37) = 2.10, *p* < .05, *d* = 1.09. No such differences were observed between Standard CBM-I and Explicit CBM-I (*t* < 1) or between CBM-I conditions and c-CBT (*t* < 1). Furthermore, the results revealed significant group differences on target sentences from pre-test to one-week follow-up between Standard CBM-I and Control, *t*(37) = 2.32, *p* < .05, *d* = 1.55, Explicit CBM-I and Control *t*(37) = 2.72, *p* < .01, *d* = 1.31, and c-CBT and Control *t*(37) = 2.07, *p* < .05, *d* = 1.09. No significant group differences were observed on foil sentences from pre-test to post-test and one-week follow-up (*t* < 1).

Taken together, these results suggest that both standard and explicit CBM-I training conditions and c-CBT increased positive interpretations of ambiguous social scenarios as compared to a control condition. Moreover, the results suggest that providing explicit instructions as compared to the standard method did not enhance the CBM-I positive effects.

### 3.2 Gain scores

Furthermore, the gain scores were subjected to one-sample *t*-tests to examine whether mean gain scores for pre-test to post-test and from pre-test to follow-up for each group were significantly different from nil differences, zero point. The results are summarised in Table 3, with both significant and non-significant findings. The one-sample *t*-tests revealed significant differences in the mean gain scores of the interpretation bias for target sentences in the Standard CBM-I, Explicit CBM-I, and c-CBT groups (*t* values from 2.16 to 4.20). However, no such differences were found for the Control group. Moreover, no significant differences were observed in the mean gain scores of the interpretation bias for foil sentences in each group (*t* values from .25 to .98). These results indicated that participants in the Standards and Explicit CBM-I groups and c-CBT group developed more positive (or less negative) interpretations of ambiguous social scenarios.

### 3.3 Attentional bias

The second aim was to demonstrate changes in attentional bias from pre- to post-test and one-week follow-up following treatment conditions. We examined the reaction time observed on the 288 attentional test trials embedded within the dot probe task to determine attentional distribution within each group of participants. Keeping with the approach adopted by MacLeod et al. (2002) to minimize the influence of outlying data points, we calculated median bias scores displayed within each experimental condition for every participant. Using the difference formula, attentional bias scores were calculated by subtracting the median response latency for trials in which the probe replaced the neutral stimuli from the median response latency for trials in which the probe replaced the emotional stimuli, such that negative numbers denote greater bias for emotional stimuli and positive numbers denote bias towards the neutral stimulus of the pair. These bias scores are shown in Table 4 for the social threat-neutral, physical sensation-neutral, and positive-neutral word pairs for each group at pre-test, post-test and follow-up.

To analyse the effects of training conditions on attentional bias the participants' bias scores were subjected to a two-way (4 × 3) ANOVA with Training Group (Standard CBM-I, Explicit CBM-I, c-CBT, Control) as a between-subjects factor and Time (pre-test, post-test, and one-week follow-up) as within-subjects factors. This analysis revealed a significant two-way interaction effect of Group × Time *F*(6,144) = 2.15, *p* < .05, indicating that after training the groups were different in terms of the response latencies to the different word types. To investigate group differences the changes in bias scores from pre-test to post-test and follow-up were subjected to one-way ANOVA. The results revealed significant groups differences from pre-test to post-test for physical sensations words, *F*(3,73) = 6.52, *p* < .05, and for social threat words, *F*(3,73) = 5.35, *p* < .05. Post-hoc comparisons with the Fisher's LSD showed significant differences on social threat words between the Standard CBM-I and Control conditions *t*(37) = 2.34, *p* < .05, the Explicit CBM-I and Control, *t*(37) = 2.18, *p* < .05, and the c-CBT and Control, *t*(37) = 2.74, *p* < .05. Furthermore, the post-hoc comparisons indicated significant differences on the physical sensation words between the Explicit CBM-I and Control condition, *t*(37) = 9.18, *p* < .01. No significant group differences were observed between bias scores from pre-test to follow-up (*t* ≤ 1).

Taken together, these results suggest that immediately after CBM-I training participants' negative attentional biases towards threat words decreased, however these positive changes were not maintained at one-week follow-up.

### 3.4 Social anxiety

Table 5 summarises the social anxiety (FNE) scores for each group at pre-test, post-test, and one-week follow-up. The means of FNE were subjected to analysis of covariance (ANCOVA) with pre-test FNE scores as covariate and the training conditions (Standard CBM-I, Explicit CBM-I, c-CBT, and Control) as between-groups factor and post-test and follow-up FNE scores as within-group factors. The result showed a significant group × time interaction effect *F*(3,73) = 4.87, *p* < .004, *η*^2^ = .17 (a medium effect size) . Post-hoc LSD revealed significant differences on FNE scores at follow-up between Standard CBM-I and Control, *t*(37) = 2.03, *p* < .05, Explicit CBM-I and Control, *t*(37) = 2.22, *p* < .05, and c-CBT and Control, *t*(37) = 3.12, *p* < .01. There was no significant between-group difference on the FNE scores at post-test *F* < 1.

Taken together, these results indicate that both the standard and explicit CBM-I paradigms and c-CBT were effective in reducing social anxiety symptom at one follow-up, although the mean values still remained above the cut-off point for FNE in all three training groups.

### 3.5 Mediation analysis

The mediation analysis was conducted to examine whether changes in either positive interpretations or attentional biases would independently mediate change in social anxiety. We entered group, change in interpretation and attentional biases, into a regression model to predict change in social anxiety at follow-up as a predicted variable. This analysis revealed that a significant interaction of group by interpretation bias scores, *R*^2^ = .46, *β* = .38, *p* < .02. The change in positive interpretations was a significant predictor of change in the FNE scores, *β* = .52, *p* < .03. The change in the attentional bias scores was not a significant predictor, *β* = .19, *p* > .20. These results suggest that only changes in positive interpretations mediated the CBM-I effect on social anxiety.

## 4 Discussion

The central feature of CBT models of social anxiety is that socially-anxious individuals have cognitive vulnerability to social anxiety due to negative information processing biases (see Clark & McManus 2002; Heinrichs & Hofmann, 2001; Hirsch & Clark 2004 for reviews). One can assume that reducing cognitive vulnerability through modifying negative cognitive biases can reduce social anxiety symptoms. Thus, the main aim of this study was to examine the effects of CBM-I positive training compared to an analogue of computer-administered CBT program and to a non-training intervention (passive control) on negative cognitive biases and social anxiety symptoms.

The first prediction was that both methods of CBM-I positive training (standard and explicit CBM-I) would decrease negative interpretations in socially-anxious individuals as compared to a control condition. The results revealed that after CBM-I high socially-anxious participants who were trained to access positive interpretations, either standard or explicit instructions, produced lower recognition ratings for negative interpretations (or higher recognition ratings for positive interpretations) of new ambiguous scenarios as opposed to a control condition. These findings suggest that both standard and explicit CBM-I training programs increased positive interpretations of the ambiguous social scenarios. These results are consistent with the previous research findings (e.g., Beard & Amir, 2008; Murphy et al., 2007) indicating that it is possible to reduce a negative interpretation bias and facilitate a positive interpretation bias of ambiguous social scenarios in socially-anxious individuals. Furthermore, the mediation analysis showed that positive endorsement of ambiguous scenarios mediated the CBM-I effects on social anxiety. This implies that CBM-I training reduced social anxiety possibly through changes in interpretation biases rather than attentional biases.

It was also hypothesised that providing the explicit instructions would enhance the CBM-I induced positive changes in interpretative bias and social anxiety as compared to the standard CBM-I training condition. The results were inconsistent with this hypothesis indicating no significant differences between standard and explicit CBM-I conditions. This is inconsistent with the view that explicit instructions to selectively process information may sometimes be unhelpful in CBM approaches (MacLeod, Martinez et al., 2009). Some studies using different variants of CBM did provide explicit instructions and still obtained beneficial effects of CBM delivery. For example, in a study by Watkins and colleagues (Watkins et al., 2009) participants with dysphoria were given explicit instructions to actively engage in generating concrete construals (e.g., focussing on the specific details of an event, on what makes each event unique, and on the process of how it happened) when imagining emotional events, as opposed to the abstract-overgeneral bias. The results showed that this *concreteness* training with explicit instructions decreased depressive symptoms and rumination in a subclinical sample. In another study, participants who were explicitly instructed to rehearse new appraisals of negative events showed a decrease in negative emotional reactions to them (Schartau et al., 2009). It is also possible that participants in the standard CBM-I training learn some rules such as making a different kind of interpretation (Brosan, Hoppitt, Sillence, Shelfer, & Mackintosh, 2011) or imagining positive outcomes (Blackwell & Holmes, 2010), which then they can apply this in their daily life. Furthermore, it is highly likely that in the standard CBM-I paradigm participants become aware of the intention of the training through repeated practice of positive resolutions of the scenarios. In a study, Salemink and colleagues found that the vast majority of their participants were aware of the emotional outcomes of the disambiguations of the passages and this awareness partially mediated the effects of training direction on interpretive bias (Salemink, van den Hout, & Kindt, 2007). In the present study, we did not measure participants' awareness of the intention of the training in standard CBM-I but similar to Salemink at al.'s study majority of the participants reported having some knowledge of the direction of the training, i.e., positive resolution of the scenarios.

One may argue that making the rule to select positive meaning explicit generated expectancy about the direction of training outcomes which can result in transitory less negative and more positive ratings of the recognition sentences (a placebo effect). Future studies using a control condition matched for expectancy and instructions, but without the ‘active’ element of CBM may shed more light on this expectancy effect. However, the fact that there was no enhanced effect of explicit instructions indicates that this effect is unlikely.

In sum, the results revealed that providing explicit instructions about the intention of the CBM-I training did not result in enhanced positive changes in interpretative bias or social anxiety. However, it is not clear whether providing explicit instructions about the applicability of CBM-I materials and practicing positive resolutions in real-life situations would enhance the effectiveness of CBM-I on reducing social anxiety. Also unlike Watkins et al.'s (2009) study, we did not instruct participants about the possible relationship between positive resolutions of CBM-I scenarios and reductions in their social anxiety. It would be interesting to investigate this further by providing more explicit instructions about the association between positive resolutions of ambiguity in real-life social situations and social anxiety.

In this study, the c-CBT program was designed as a single session of short, condensed computerised CBT and used as an alternative intervention to compare CBM-I with an analogue of computerised CBT. Consistent with the second hypothesis, the results also showed that a single-session computer-administered CBT for social anxiety increased positive interpretations of ambiguous scenarios in socially-anxious participants. This is consistent with the finding reported by Franklin and colleagues that socially-phobic individuals treated with 14-weekly group CBT exhibited less negative interpretation bias than untreated socially-phobic individuals (Franklin et al., 2005). This finding is promising because it indicates that similar to a long-term CBT treatment, a computer-administered CBT program can generate more positive (or less negative) interpretations of ambiguous social situations. One possible explanation for positive changes in interpretations biases via c-CBT is that this program increased participants' awareness of the role of negative thinking pattern (e.g., negative automatic thoughts, dysfunctional assumptions) in social anxiety which in turn resulted in more positive interpretations of the ambiguous social scenarios.

It was assumed that the CBM-I training would reduce selective attention to threatening stimuli. Consistent with this hypothesis, the results showed that, to some degree, CBM-I reduced negative attentional biases towards threat information. These results are in line with the findings of two previous studies (Amir, Bomyea, & Beard, 2010; White et al., 2011) demonstrating that modifications of one component of information processing, i.e., attention or interpretation, can also result in changes in the other component. These findings suggest that targeting interpretation biases through CBM-I positive training may also have some positive immediate effects on modifying attention bias. However, it should be noted that despite some significant changes in attentional bias at post-test, the bias scores were not large in this sample and changes in the bias scores were not sustained at one-week follow-up. Future studies should investigate the effects of CBM-I on attentional bias further possibly by comparing CBM-I with an attentional variant of CBM (CBM-A).

Moreover, the results revealed a significant reduction in social anxiety with a medium effect size for the CBM-I and c-CBT conditions at one-week follow-up. This is consistent with a previous finding reported by Beard and Amir (2008) that changes in interpretation bias reduced social anxiety symptoms. However, the fact that despite positive changes in the FNE scale at follow-up, these scores remained above the cut-off point (clinical significance) for the training conditions suggest that a multi-sessions CBM-I training might produce a larger effect size which is clinically significant. It remains possible that genuine CBM-I or c-CBT induced changes in cognitive biases might exert an impact that does not necessarily concur with immediate changes in self-report questionnaires as these measures assess mainly more prolonged behavioural and emotional features.

Given the findings of the present study, it is important for future CBM-I research to pursue new directions. One of the limitations of the present study was that the recognition test as a measure of interpretation bias has not yet been validated for social anxiety. To address this, the future research should focus on comparing two distinct groups of people with clinical social anxiety and without social anxiety on interpretations biases. This design can give more information about the validity of this measure. Another limitation of this study was that it was designed as a single session of CBM or an analogue session of c-CBT. Despite some promising effects of CBM-I and c-CBT in reducing negative cognitive biases in social anxiety, it would be naive to expect that direct modification of cognitive biases through a single session will yield enduring therapeutic benefits. One would expect that multi-sessions CBM-I or c-CBT programs would induce more durable changes in interpretation biases and social anxiety symptoms.

Moreover, multiple practices outside of the laboratory setting (e.g., using home computer) may prove to result in more enduring therapeutic effects on both cognitive biases and social anxiety symptoms. This may be of a particular interest when applying CBM-I to a clinical population. As MacLeod, Koster, and Fox (2009) suggested, future researchers should investigate how to optimise the transfer of CBM-induced cognitive changes from the laboratory into the naturalistic setting and to effectively maintain these modified selective processing across time. This is important for both theoretical and clinical reasons. From a theoretical point of view, it is important to determine whether CBM-I induced changes fundamentally alter cognitive processes that cause and/or maintain social anxiety or if they simply produce transient, context-dependent effects. It is also clinically important to determine whether the CBM-induced changes can affect deeper levels of information processing involved in maintaining social anxiety symptoms such as negative self-appraisal (see Mobini et al., 2013). Finally, more research is needed to provide evidence for clinical efficacy and effectiveness of CBM-I as compared to other well-established treatments such as CBT (NICE, 2013) and whether CBM-I can be used as an adjunct treatment method with CBT for social anxiety.

## 5 Conclusions

Taken together, this study suggests that a single session of CBM-I, using either standard or explicit instructions, and an analogue of c-CBT program increased positive interpretations of ambiguous scenarios and reduced social anxiety symptoms. Furthermore, CBM-I programs reduced attentional biases towards threat information. However, the durability of these modified cognitive biases and changes in underlying cognitive processing (e.g., self-appraisals) maintaining social anxiety remain to be seen. Further research on CBM-I is of particular value in investigating the efficacy of this intervention in the treatment of social anxiety using rigours clinical trials.

## Figures and Tables

**Table 1:** Demographic information for each group.

	Standard CBM-I (*n* = 19)	Explicit CBM-I (*n* = 19)	c-CBT (*n* = 19)	Control (*n* = 19)
Age M(SD)	25 (12)	21 (3)	25 (9)	23 (8)
Gender (female)	13 (68%)	14 (74%)	15 (79%)	14 (74%)
Ethnicity	18 Caucasian 1 African	17 Caucasian 1 White/Caribbean 1 Asian	15 Caucasian 1 White/Caribbean 1 Asian/White 2 Asian	16 Caucasian 2 Asian 1Asian/White

**Table 2:** Means (*M*) and standard errors (SE) of bias scores for the target and foil sentences for each group at pre-test, post-test, and one-week follow-up (*N* = 76). Negative scores represent less positive interpretations of new ambiguous social scenarios and positive scores represent more positive interpretations of these scenarios.

	Standard CBM-I	Explicit CBM-I	c-CBT	Control
Pre-test				
Targets	−.65 (.13)	−.42 (.19)	−.42(.17)	**−.47** (**.22)**
Foils	.10 (.07)	.13 (.11)	.17 (.12)	**.09** (**.14)**
Post-test				
Targets	.15* (.13)	.53* (.18)	.14* (.13)	**−.32** (**.22)**
Foils	.14 (.07)	.22 (.10)	.33 (.16)	**.03** (**.10)**
Follow-up				
Targets	.28* (.14)	.48** (.21)	.34* (.12)	**−.37** (**.22)**
Foils	.10 (.06)	.19 (.08)	.23 (.13)	**.09** (**.15)**

Note: Significant differences are shown between each group (Standard CBM-I, Explicit CBM-I, c-CBT and Control) and the Control Condition (Bold). *p < .05, **p < .01.

**Table 3:** Means (*M*) and Standard Error (SE) of the gain scores on the interpretation bias measure for each group from pre-test to post-test and from pre-test to follow-up.

	Standard CBM-I	Explicit CBM-I	c-CBT	Control
Pre-test to post-test				
Targets	.80** (.23)	.95*** (0.23)	.49* (.22)	.15 (.12)
Foils	.04 (.10)	.08 (.16)	.16 (.14)	.06 (.10)
Pre-test to follow-up				
Targets	.93** (.22)	.90** (.26)	.71** (.21)	.10 (.17)
Foils	.005 (.10)	.06 (.12)	.06 (.16)	.003 (.15)

Note: Significance levels (*p* values) indicate the differences of each gain score from zero, one-sample t-test, two-tailed. *p < .05, **p < .01, ***p < .001.

**Table 4:** Means (*M*) and standard errors (SE) of bias scores for the social threat, physical sensations, and positive word pairs for each group at pre-test, post-test, and one-week follow-up (*N* = 76). Negative scores represent a bias towards the emotional stimulus of the pair.

Work type	Standard CBM-I	Explicit CBM-I	c-CBT	Control
Pre-test				
Threat	−6.62 (6.53)	−10.24 (5.26)	−3.61 (4.26)	**.39** (**3.29)**
Physical	3.21 (1.16)	−12.82 (6.05)	−7.03 (4.55)	**−1.66** (**3.44)**
Positive	−.21 (2.86)	.38 (3.36)	2.25 (4.92)	**−4.50** (**2.94)**
Post-test				
Threat	6.50* (3.62)	3.50* (3.55)	4.58* (3.60)	.**29** (**4.27)**
Physical	.91 (4.53)	9.00** (2.98)	1.33 (3.17)	**−4.76** (**2.43)**
Positive	.68 (3.43)	1.53 (3.40)	1.34 (4.21)	**−2.29** (**2.29)**
Follow-up				
Threat	.18 (4.51)	1.47 (4.11)	.39 (4.33)	**−1.71** (**2.38)**
Physical	1.44 (2.63)	4.50 (2.14)	3.86 (3.11)	**−1.32** (**3.96)**
Positive	1.94 (3.35)	−.15 (2.71)	−.53 (3.74)	**−2.92** (**2.96)**

Note: Significant differences are shown between each group (Standard CBM-I, Explicit CBM-I, c-CBT and Control) and the Control Condition (Bold). *p < .05, **p < .01.

**Table 5:** Means and standard deviations of the FNE scale for each group at pre-test and one-week follow-up (*N* = 76).

	Standard CBM-I	Explicit CBM-I	c-CBT	Control
Pre-test	23.26 (3.69)	24.06 (3.53)	23.79 (3.92)	**24.39** (**3.24)**
Post-test	23.21 (3.79)	23.44 (3.36)	22.89 (4.77)	**23.56** (**5.03)**
Follow-up	20.89* (3.56)	19.94* (3.24)	18.53** (3.50)	**23.72** (**3.70)**

Note: Significant differences are shown between each group (Standard CBM-I, Explicit CBM-I, c-CBT and Control) and the Control Condition (Bold). *p < .05, **p < .01.
